# Stat1 Nuclear Translocation by Nucleolin upon Monocyte Differentiation

**DOI:** 10.1371/journal.pone.0008302

**Published:** 2009-12-14

**Authors:** Uwe Jerke, Sergey Tkachuk, Julia Kiyan, Victoria Stepanova, Angelika Kusch, Michael Hinz, Rainer Dietz, Hermann Haller, Bianca Fuhrman, Inna Dumler

**Affiliations:** 1 Hannover Medical School, Hannover, Germany; 2 Experimental and Clinical Research Center-ECRC, at the Max Delbrück Center, Berlin, Germany; 3 Department of Pathology and Laboratory Medicine, University of Pennsylvania, Philadelphia, Pennsylvania, United States of America; 4 Lipid Research Laboratory, Technion Faculty of Medicine, Rappaport Institute for Medical Sciences, and Rambam Medical Center, Haifa, Israel; 5 Max Delbrück Center, Berlin, Germany; Bauer Research Foundation, United States of America

## Abstract

**Background:**

Members of the signal transducer and activator of transcription (Stat) family of transcription factors traverse the nuclear membrane through a specialized structure, called the nuclear pore complex (NPC), which represents a selective filter for the import of proteins. Karyophilic molecules can bind directly to a subset of proteins of the NPC, collectively called nucleoporins. Alternatively, the transport is mediated via a carrier molecule belonging to the importin/karyopherin superfamily, which transmits the import into the nucleus through the NPC.

**Methodology/Principal Findings:**

In this study, we provide evidence for an alternative Stat1 nuclear import mechanism, which is mediated by the shuttle protein nucleolin. We observed Stat1-nucleolin association, nuclear translocation and specific binding to the regulatory DNA element GAS. Using expression of nucleolin transgenes, we found that the nuclear localization signal (NLS) of nucleolin is responsible for Stat1 nuclear translocation. We show that this mechanism is utilized upon differentiation of myeloid cells and is specific for the differentiation step from monocytes to macrophages.

**Conclusions/Significance:**

Our data add the nucleolin-Stat1 complex as a novel functional partner for the cell differentiation program, which is uniquely poised to regulate the transcription machinery via Stat1 and nuclear metabolism via nucleolin.

## Introduction

Human blood monocytes are able to differentiate into morphologically and functionally heterogeneous effector cells, including macrophages. The precise molecular mechanisms responsible for differentiation of circulating monocytes into tissue macrophages are, however, incompletely defined. Recent studies highlight the role of transcription factors and other nucleo-cytoplasmic shuttling proteins in these processes, which require dynamic changes in gene expression [1], [2].

Nucleolin is an ubiquitous multifunctional nucleolar shuttle phosphoprotein in eukaryotic cells. Its tripartite domain structure, with an acidic histone-like N-terminus, a central domain containing four RNA binding domains, and an arginine and glycine rich C-terminus, reflects the diverse roles of nucleolin in cell growth, proliferation, and cell death (reviewed in [3]–[5]). Nucleolin has been implicated in many cellular activities, including pre-ribosomal RNA transcription and ribosome biogenesis [6], replication and recombination of DNA, cell cycle progression [7], viral infection [8], [9], and apoptosis [10]–[13]. One remarkable characteristic of nucleolin is that it shuttles constantly between the nucleus and the cytoplasm [14] and additionally serves in some cell types as a cell surface receptor [12], [15]–[17]. For the nucleocytoplasmic translocation, nucleolin uses its bipartite nuclear localisation signal (NLS) located between the N-terminal and central domains, and thereby acts as a carrier for karyophilic proteins [18]–[20]. A growing body of evidence shows interactions of nucleolin with transcription factors [6], [21]–[23].

Members of the signal transducer and activator of transcription (Stat) family of transcription factors are activated during the myeloid differentiation and may play an important role in the differentiation program, including those of monocyte-to-macrophages [24]–[26], [2], [27], [28]–[30]. In response to ligand binding of cytokines and growth factors to cell surface receptors, the cytoplasmically located Stats become phosphorylated, form dimers, enter the nucleus, and bind to specific DNA sequences that often results in an alteration of gene expression profiles [31]. The exact role of Stat proteins in the regulation of proliferation and terminal cell differentiation of myeloid cells remains to be elucidated.

To enter the nucleus, the Stats have to traverse the nuclear membrane through a specialized structure, called the nuclear pore complex (NPC), which represents a selective filter for the import of proteins [32]. Karyophilic molecules can bind directly to a subset of proteins of the NPC, collectively called nucleoporins [33], [34]. Alternatively, the transport is mediated via a carrier molecule belonging to the importin/karyopherin superfamily, which binds to the NLS of the macromolecular cargoes, and transmits the import into the nucleus through the NPC. For Stat proteins both, the carrier-independent and the carrier-dependent nucleocytoplasmic shuttling have been described [35], [36].

The aim of the present work was to analyze a possible involvement of the multifunctional shuttle protein nucleolin in myeloid differentiation of monocytic cells to macrophages. We report that during the monocyte-to-macrophage differentiation, nucleolin associates with the transcription factor Stat1. This association is specific for cells of monocytic origin and is involved in the monocyte-to-macrophage differentiation program. Using expression of nucleolin transgenes, we found that the NLS sequence of nucleolin is responsible for Stat1 nuclear translocation and formation of a ternary complex of nucleolin, Stat1 and the Stat1 target DNA. Our study provides evidence that in addition to so far known Stat1 nuclear import mechanisms an alternative pathway exist, which involves nucleolin-mediated Stat1 transport to the nucleus.

## Results

### Nucleolin and Stat1 Associate during Monocyte-to-Macrophage Differentiation

**Figure 1:**
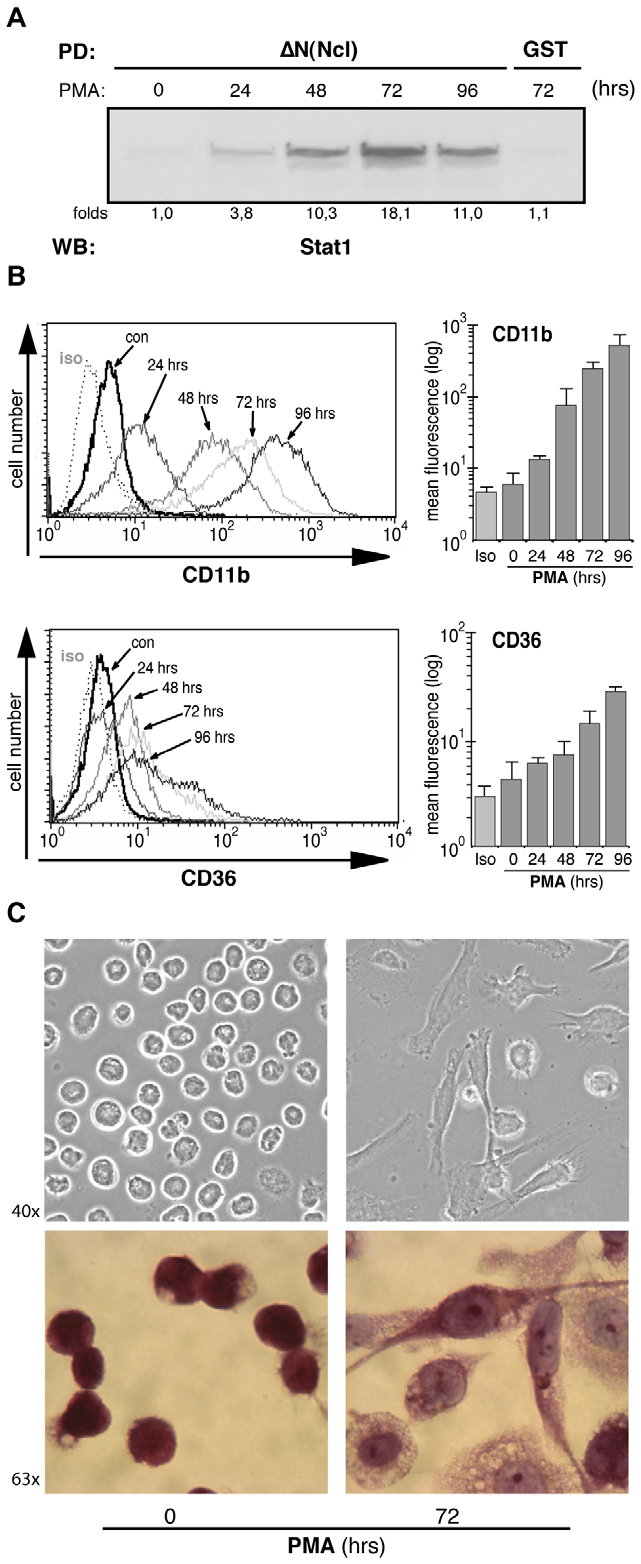
Stat1 and nucleolin bind in THP-1 cells in a time-dependent manner. (A) Nucleolin-GST fusion proteins were bound to glutathione agarose and used as affinity matrix in a pull-down assay with whole cell extracts from non-stimulated or PMA (2 nM) stimulated THP-1 cells. Eluates were analyzed by Western blotting; GST glutathione agarose was used as control. (B) THP-1 cells treated as described above were analyzed by FACS analysis. Cell surface expressions of CD11b and CD36 were used to monitor the process of the monocyte-macrophage differentiation. (C) Phase contrast picture and Wright-Giemsa staining of non-stimulated and PMA stimulated THP-1 cells.

Nucleolin plays an important role in the differentiation program of hematopoietic cells [37], [38]. As a nucleocytoplasmic shuttle protein, nucleolin binds to transcription factors and modulates gene expression during differentiation. To identify binding partners of nucleolin involved in monocytes differentiation, a nucleolin affinity matrix precipitation assay was performed. For this purpose, we used the N-terminal domain lacking nucleolin construct (ΔN-Ncl) generated from human cDNA clones, bacterially expressed as a GST fusion protein and bound to glutathione agarose. Human myeloid leukaemia THP-1 cells were stimulated up to four days with the phorbol ester PMA to induce the differentiation process, and whole cell lysates were used for the pull-down assay. It has been reported previously that Stat proteins are activated during myeloid differentiation [2]. Therefore, we addressed first these transcription factors and analyzed eluates of our pull-down assays using antibodies against Stat proteins. We found in these experiments that the transcription factor Stat1, but not other Stats (Stat2, Stat3, Stat4, Stat5), was specifically bound to the nucleolin-GST matrix (Fig. 1A and data not shown). This binding was time-dependent peaking at 72 hrs of PMA treatment. The macrophage differentiation was monitored by the expression of specific cell surface proteins (Fig. 1B). In the phase contrast images and after Wrights-Giemsa staining, PMA treated cells showed the characteristic macrophage morphology, namely, larger cell size, increased membrane ruffles, and large cytoplasm pseudopodia of the adherent cells (Fig. 1C).

**Figure 2:**
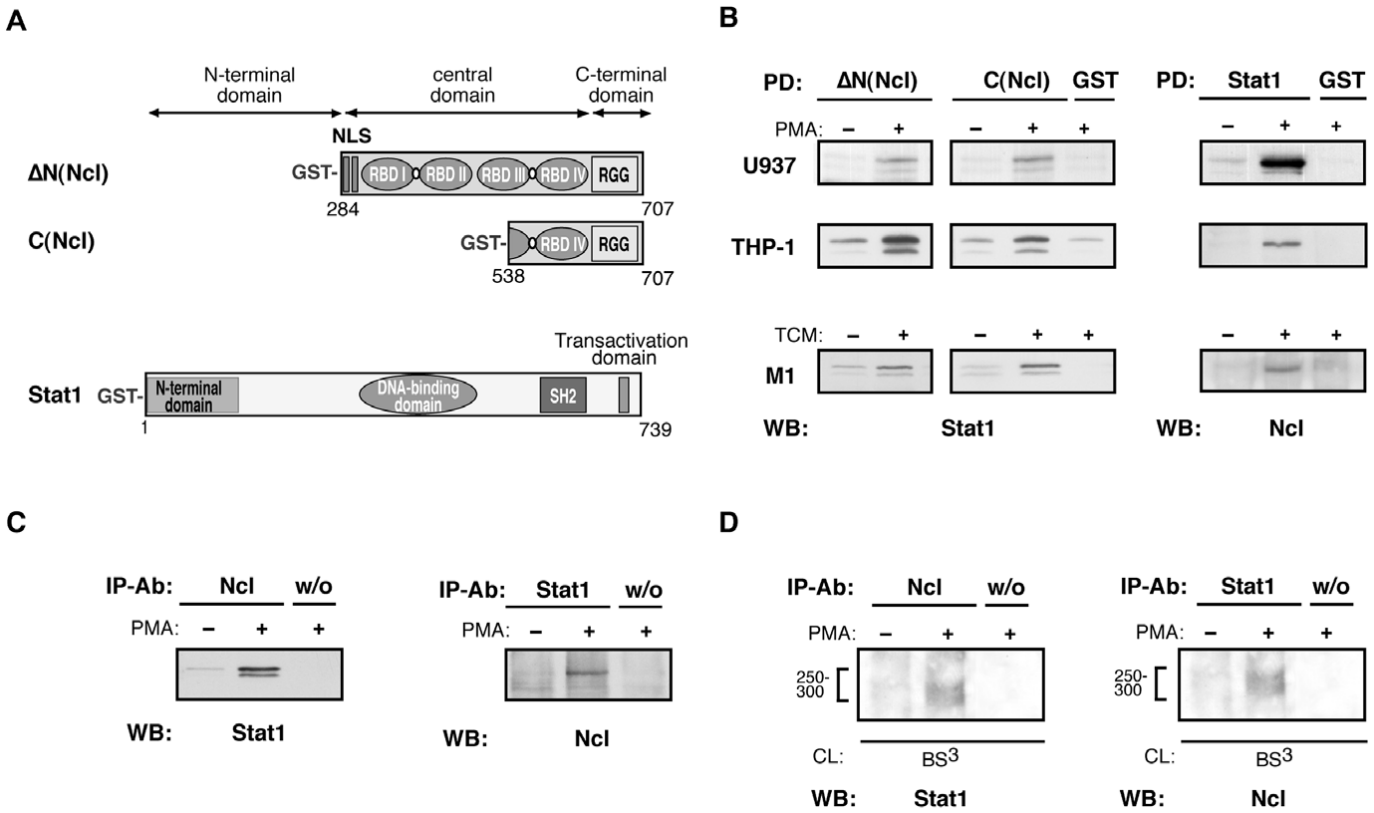
Stat1-nucleolin complex is specifically formed in cells of monocytic origin. (A) Schematic presentation of nucleolin and Stat1 constructs generated from human cDNA clones to express GST fusion proteins. (B) Different cell lines of monocytic origin cultured for 72 hrs without or with indicated stimuli were analyzed in pull-down assay using nucleolin- or Stat1-GST fusion proteins bound to glutathione agarose. Respective anti-Stat1 or anti-Ncl antibody was used for Western blotting of eluates. (C) Co-immunoprecipitation studies with whole-cell extracts from non-stimulated or PMA stimulated THP-1 cells. Anti-nucleolin or anti-Stat1 antibody coupled to protein A/G-agarose was used as affinity matrix. Eluted proteins were analyzed by Western blotting with corresponding antibodies to detect Stat1 or nucleolin. (D) Proteins were released by hypotonic shock of non-stimulated or PMA stimulated THP-1 cells. S100 fractions were subjected to chemical cross-linking using BS^3^, followed by immunoprecipitation and Western blotting.

To provide further evidence for the nucleolin-Stat1 binding, several approaches were used. Since the C-terminal glycine/arginine-rich domain of nucleolin (RGG) was reported to serve for nucleolin interactions [3], the corresponding nucleolin constructs (C(Ncl) and ΔN(Ncl)) were generated, as well as a full-length Stat1 (Fig. 2A). Pull-down assays using both ΔN(Ncl)-GST and C(Ncl)-GST, as well as Stat1-GST, confirmed nucleolin- Stat1 binding in THP-1 cells stimulated for differentiation. These results suggest that the RGG region of nucleolin is involved in this interaction. The Stat1-nucleolin binding was additionally found in cells of the human U937 promyelocytic cell line and in the M1 mouse myeloid leukemia cells stimulated for differentiation (Fig. 2B). To assess whether nucleolin and Stat1 might associate in living cells, co- immunoprecipitation experiments using anti-nucleolin and anti-Stat1 antibodies were performed. Indeed, both proteins were co-immunoprecipitated. Remarkably, nucleolin-Stat1 association was only observed in cells stimulated for differentiation, but not in the unstimulated cells (Fig. 2C). As a third approach investigating Stat1-nucleolin interference, we performed cross-linking studies (Fig. 2D). Cytosolic proteins of PMA stimulated THP-1 cells were subjected to cross-linking using chemical cross-linker BS^3^, followed by immunoprecipitation with anti-nucleolin (left panel) or anti-Stat1 antibodies (right panel). An additional high molecular mass band over 250 kDa was revealed in both experimental settings. From the molecular mass value, we assume that the complex contains a Stat1 dimer and nucleolin. These findings implicate a direct interaction between Stat1 and nucleolin.

**Figure 3:**
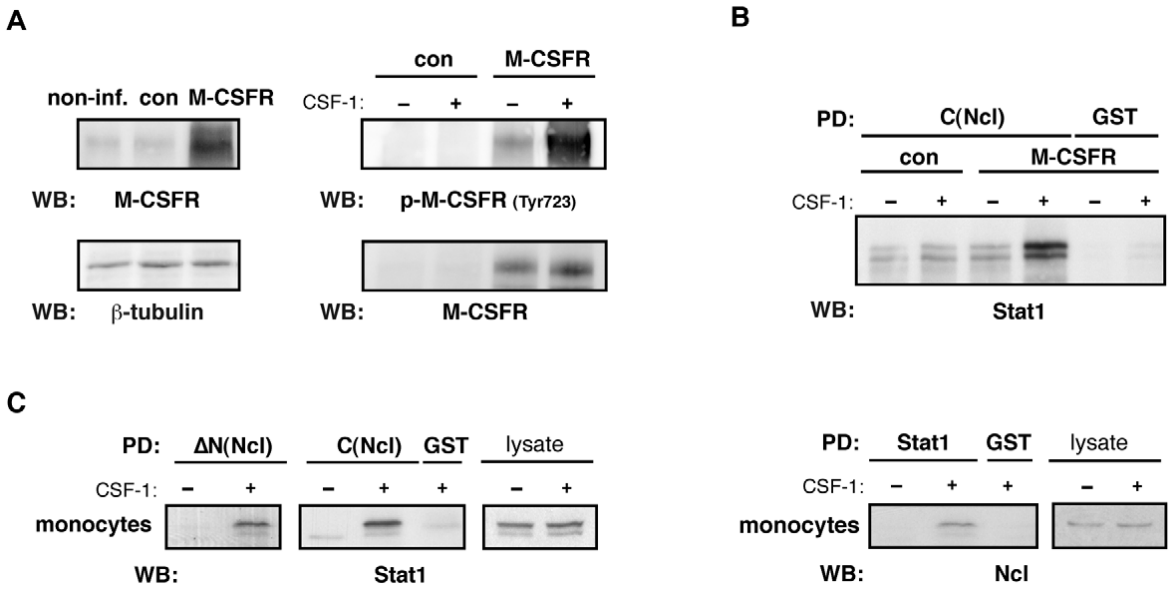
Formation of the Stat1-nucleolin complex in human monocytes is mediated by the M-CSFR. (A) Lysates of non-, control- or M-CSFR- infected THP-1 cells were immunoblotted with anti-M-CSFR antibody (upper left panel). Autophosphorylation of M-CSFR in M-CSFR-expressing THP-1 cells after stimulation with CSF-1 was detected by phospho-specific anti-M-CSFR IgG (upper right panel). (B) M-CSFR- expressing THP-1 cells were stimulated with CSF-1 and taken for pull-down experiments. (C) Human peripheral blood-derived monocytes (Mo) were stimulated with CSF-1 and used for pull-down assay as indicated in above. Crude cell lysates were included to demonstrate equal protein expression of Stat1 and Ncl after CSF-1 stimulation.

Since usage of cell lines and cell stimulation with PMA reflects a model system, we were interested whether association of nucleolin with Stat1 might have a physiological relevance. For this purpose, a full-length human macrophage colony stimulating factor receptor (M-CSFR), which is a physiological receptor to mediate monocyte differentiation, was expressed in THP-1 cells by means of a lentiviral gene transfer. The expressed M-CSFR was functionally competent and revealed an autophosphorylation in response to stimulation with its natural ligand, CSF-1 (Fig. 3A). Pull-down assay demonstrated binding of nucleolin and Stat1 in M-CSFR expressing THP-1 cells stimulated with CSF-1 (Fig. 3B). The same results were obtained in highly purified human peripheral blood derived monocytes (Fig. 3C), but not in cells of non-monocytic origin like primary vascular smooth muscle cells, endothelial cells or cells of the fibrosarcoma HT1080 cell line (data not shown). The increased association of Stat1 with nucleolin upon cell stimulation was not related to changes in their expression. As shown in control experiments, neither Stat1 nor nucleolin protein expression was affected upon the differentiation process (Fig. 3C).

Together, these data indicate that Stat1-nucleolin association is a physiological phenomenon specific for cells of monocytic origin.

### Nucleolin-Stat1complex Translocates into the Nucleus and Binds to the Stat1 Specific DNA Sequence GAS

**Figure 4:**
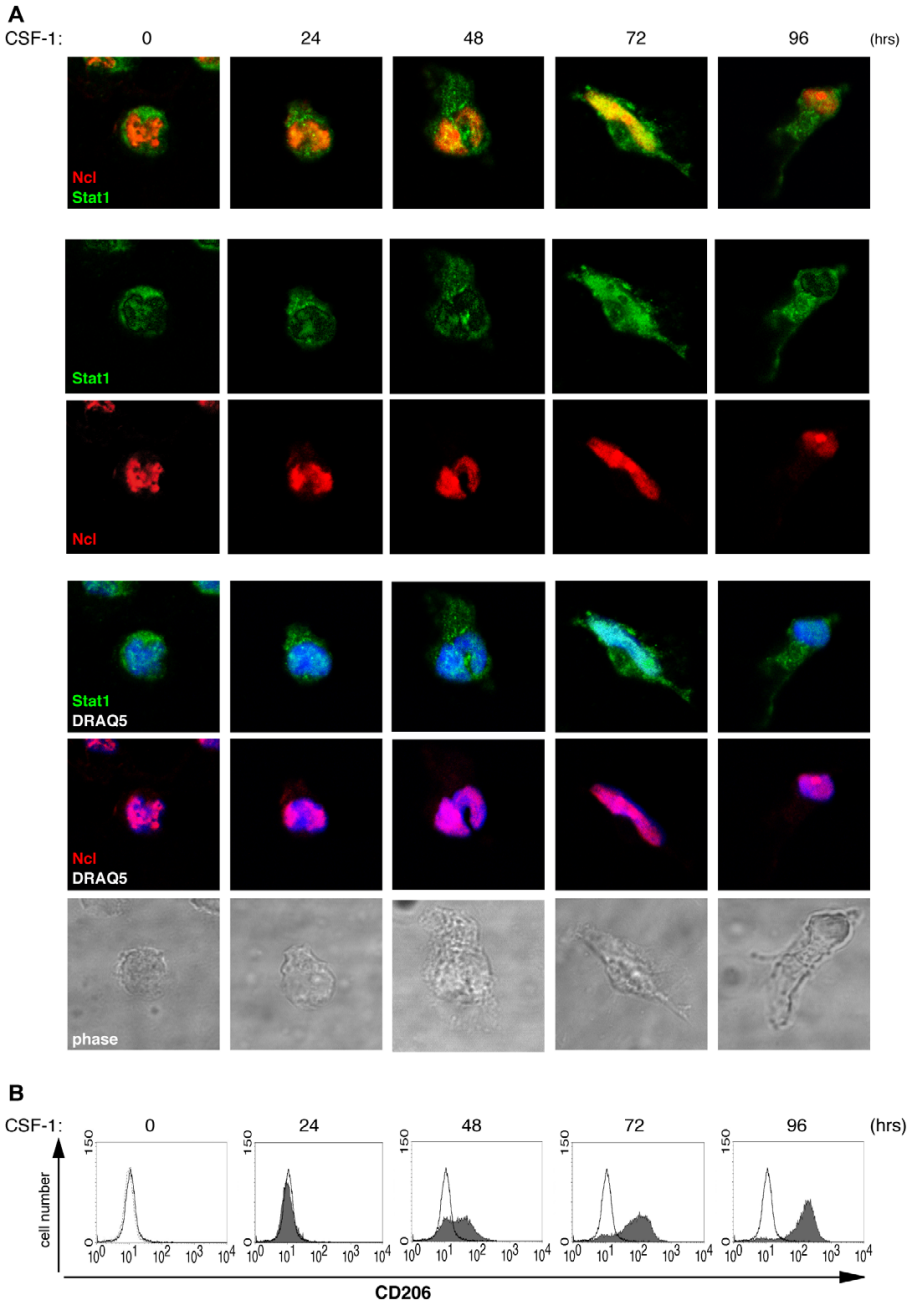
Stat1 and nucleolin are translocated into the nucleus. (A) Primary blood-derived human monocytes were fixed, permeabilized, and stained using polyclonal anti-nucleolin antibody, monoclonal anti-Stat1 antibody, and the corresponding Alexa coupled secondary antibodies; simultaneous DNA labelling with DRAQ5 was performed to visualize the nuclear compartment. The merged confocal images of monocytes stimulated with CSF-1 for indicated time points are shown. The lower panel shows phase contrast pictures. The images were acquired with a resolution of 1024x1024 pixels with a MRC1024 confocal microscope (BioRad, Hercules, CA) attached to a Nikon Diaphot. All images were taken with oil-immersed x63 objective, and were recorded for triple staining sequentially with detection wavelengths range for Alexa488, Alexa568 and DRAQ5 (Exλmax 646 nm). Pictures were merged using the Lasersharp software (BioRad). (B) The monocyte-to-macrophage differentiation was monitored by FACS analysis. Cell surface expression of the macrophage-mannose recepor (CD206) in CSF-1 stimulated (dark grey curve) and non-stimulated monocytes (open line) are shown. The broken line represents the FITC-isotype control.

Nucleolin can shuttle between the nucleus and the cytoplasm [14]. Activated Stat proteins form dimers, enter the nucleus, and bind to specific DNA sequences to affect gene transcription [31]. To investigate the cellular localization of nucleolin and Stat1 during the differentiation process, immunocytochemical studies were performed. Human primary monocytes were stimulated with CSF-1 for indicated time points to induce monocyte-to-macrophage differentiation. The staining patterns in the confocal images demonstrated that though Stat1 was mainly expressed in the cytoplasm and nucleolin in the nucleus, both proteins revealed co-localization in the perinuclear space followed by their nuclear translocation. The maximal colocalization of nucleolin and Stat1, as well as individual colocalization of both proteins with nuclear marker was observed after 72 hours CSF-1 treatment. At this time point, most of the cells showed the characteristic macrophage morphology (Fig. 4A, lower panel). Cell differentiation was additionally controlled by the expression of the cell surface macrophage-mannose receptor (CD206) by FACS analysis (Fig. 4B). The observed nucleolin-Stat1 colocalization was transient and decreased with longer stimulation.

**Figure 5:**
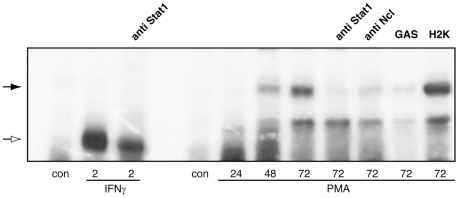
PMA induces binding of a Stat1-nucleolin complex to the GAS element. THP-1 cells were treated with IFNγ for 2 h or with PMA for 24, 48 or 72 h. Whole cell extracts were prepared and used for EMSA. Subunit composition of DNA binding complexes were analyzed by antibody competition with antibodies directed against Stat1 and nucleolin, as indicated. Complex specificity was verified with oligonucleotide competition, using 5-fold excess of GAS or H2K, respectively. White arrow indicates Stat1 homodimers; black arrow indicates a Stat1-nucleolin complex.

We next asked whether or not the nucleolin-Stat1 complex binds to the Stat1 specific DNA sequences after translocation into the nucleus. In our EMSA, ^32^P-labeled GAS oligonucleotide was used as a probe to analyze nuclear extracts from THP-1 cells activated with PMA for up to 72 hours. Cells stimulated with interferon-gamma (IFNγ), which is one of the most effective Stat1-activating cytokines, were used as a positive control. The results of these experiments are presented in Fig. 5. Cell stimulation with PMA led to the induction of one specific DNA-binding protein complex migrating in gel more slowly than complex formed in response to IFNγ. The kinetics of the DNA binding activity correlated with those for the Stat1-nucleolin nuclear translocation (Fig. 4A). No binding to the GAS was detected in the presence of excess of unlabeled GAS, whereas an unrelated oligonucleotide did not affect the DNA-protein binding. To examine the presence of Stat1 and/or nucleolin in the observed complex, a supershift assay was performed. The corresponding band was inhibited by both anti-Stat1 and anti-nucleolin antibody.

These data implicate a role of the nucleolin-Stat1 complex for the regulated gene expression upon the monocyte differentiation program.

### Nucleolin Serves as a Carrier for the Nuclear Translocation of Stat1

**Figure 6:**
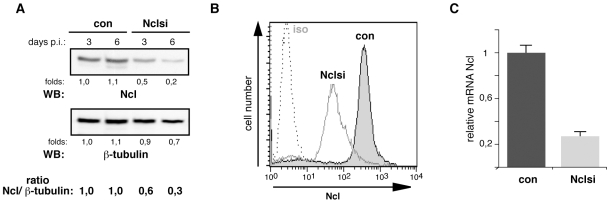
Nucleolin gene silencing in THP-1 cells. (A) For each experiment, the nucleolin downregulation in the THP-1 by lentiviral gene silencing was checked on protein level 3 and 6 days post infection by Western blotting of whole cell extracts with monoclonal anti-nucleolin antibody (upper panel; lower panel demonstrates equal protein loading) or by FACS analysis of fixed and permeabilzed cells (B) or on mRNA level by quantitative RT-PCR analysis (C).

Molecular mechanisms of Stat1 nuclear translocation have been intensively studied [39] However, an involvement of nucleolin in this process has not been reported yet. Therefore, we next examined whether the observed association of nucleolin and Stat1 is necessary for the transport of this transcription factor into the nucleus. We specifically inhibited nucleolin expression in THP-1 cells by RNA silencing using a lentiviral RNA interference vector constructed for this purpose. Up to 70% of infection rate was achieved by this way (Fig. 6), whereas conventional transfection reagents showed only low efficiency in cells of monocytic origin [40].

**Figure 7:**
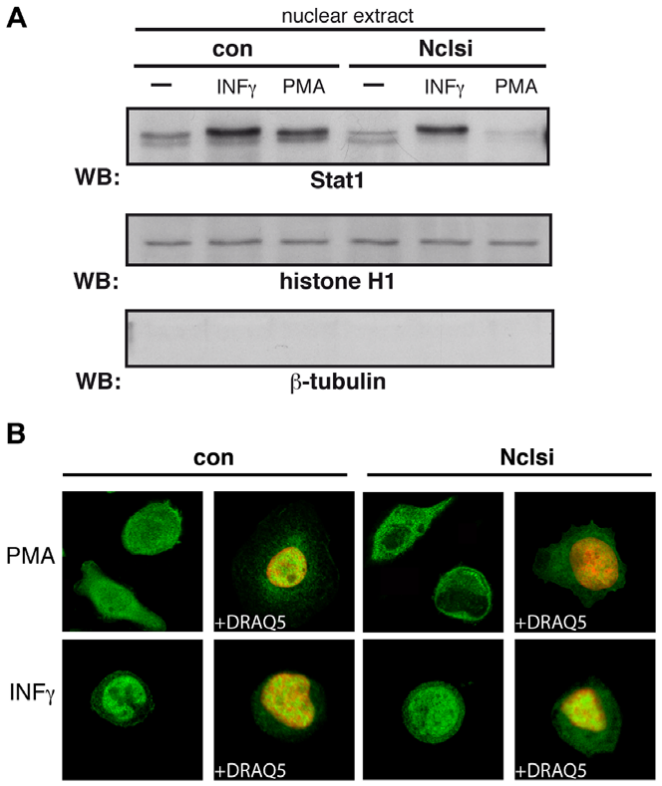
Nucleolin gene silencing prevents nuclear Stat1 translocation. (A) THP-1 cells were infected with nucleolin-si lentiviruses (Nclsi) or empty viruses (con). Three days post infection, cells were cultivated for further 72 hrs without or with 2 nM PMA or stimulated with INF-γ for 2 hrs. Nuclear extracts were prepared, separated by SDS-PAGE, and Stat1 nuclear translocation was visualized by Western blotting using monoclonal anti-Stat1 antibody (upper panel). The purity of cell fractionation was proved by reprobing the membrane with anti-histone antibody (nuclear marker, middle panel) and anti-β-tubulin IgG (cytoplasmic marker, lower panel). (B) Inhibition of the Stat1 nuclear translocation is shown by confocal microscopy studies in Nclsi infected THP-1 cells stimulated as indicated. Cells were fixed and permeabilized, and monoclonal anti-Stat1 antibody and corresponding Alexa 488-coupled secondary IgG were used for staining (green colour). Nuclear compartments were visualized by simultaneous DNA labelling with DRAQ5 (red colour). The fluorescence cell images were captured using a Leica TCS-SP2 AOBS confocal microscope (Leica Microsystems). All images were taken with oil-immersed x63 objective, NA = 1.4. Resolution 1024×1024.

To elucidate whether nucleolin is required for the nuclear transport of Stat1, we performed cell fractionation and examined nuclear extracts isolated from Nclsi-THP-1 cells, non-treated or treated with PMA for 72 hrs. Specific markers were used to control the purity of obtained fractions (Fig. 7A). Stat1 enrichment in nuclear fractions was strongly impaired in PMA stimulated Nclsi-THP-1 cells, but not in control infected cells. Interestingly, we found no impact of nucleolin on the Stat1 nuclear transport in response to IFNγ. Thus, although IFNγ elicited Stat1 enrichment in nuclear fractions, there was no difference between the IFNγ stimulated Nclsi-THP-1 cells and control infected cells. In addition, longer stimulation with IFNγ did not show any influence of nucleolin on the Stat1 nuclear distribution (data not shown). These data favor a specificity of the nucleolin- dependent Stat1 nuclear translocation for the monocyte-to-macrophage differentiation, which is independent of IFNγ.

To verify these findings further, we used Nclsi-THP-1 cells under the same experimental design for confocal microscopy studies (Fig. 7B). The staining patterns for Stat1 were consistent with the results of immunoblotting of the nuclear extracts (Fig. 7A). Thus, Stat1 nuclear translocation in response to PMA was blocked in nucleolin down-regulated cells, whereas in control-infected cells Stat1 was effectively distributed to the nucleus of differentiating cells (Fig. 7B, upper panels). Independently of the virus construct and stimulation time used, IFNγ stimulated THP-1 cells showed a clear translocation of Stat1 into to nucleus (Fig. 7B, lower panels, shown for 2 hrs IFNγ stimulation).

Together, these findings demonstrate the requirement of nucleolin for the nuclear transport of the transcription factor Stat1 upon the monocyte differentiation process.

### The Stat1-Nucleolin Interference Is Specific for the Monocyte-to-Macrophage Differentiation Step and Regulates the Expression of the Macrophage Scavenger Receptor CD36

**Figure 8:**
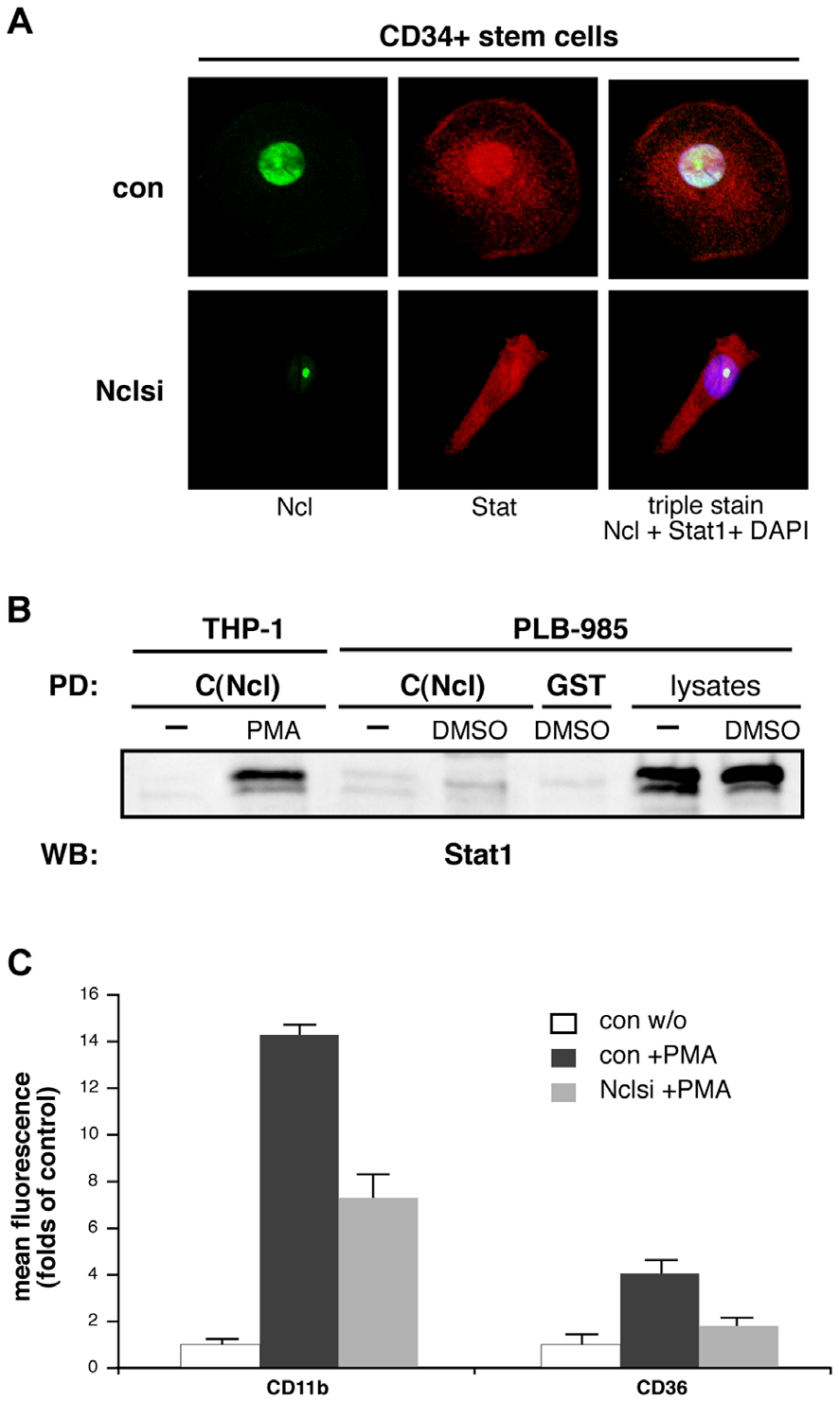
Stat1-nucleolin complex is specific for the differentiation step of monocytes to macrophages and regulates expression of the macrophage scavenger receptor CD36. (A) Peripheral blood CD34+ cells were stimulated with M-CSF, to induce differentiation to the monocyte/macrophage specific unilineage. Nucleolin downregulation in the CD34+ cells was achieved by lentiviral gene silencing. Cells were fixed, permeabilized, and stained using polyclonal anti-nucleolin antibody (green colour), monoclonal anti-Stat1 antibody (red colour), and the corresponding Alexa-coupled secondary antibodies; simultaneous DNA labelling with DAPI (blue colour) was performed to visualize the nuclear compartment. The merged confocal images of Ncl-Stat1-DAPI triple stained CD34+ cells taken by a Leica TCS-SP2 AOBS confocal microscope are shown in the right panel. (B) Nucleolin-GST pull-down assay with whole cell extracts from monocytic THP-1 cells and PLB-985, differentiated to a neutrophilic phenotype by DMSO treatment. Eluates were analyzed by Western blotting; GST glutathione agarose was used as control. Crude cell lysates show equal Stat1 expression in PLB-985 cells. (C) THP-1 cells, lentiviral Nclsi-pLVTHM (Nclsi) infected or with empty lentiviruses (con) treated, were analyzed by FACS after induction of monocyte/macrophage differentiation by PMA. Cell surface expressions of CD11b and CD36 are shown.

Nucleolin is highly expressed in cells of the hematopoietic system and its important role for basic biological functions in hematopoietic stem/progenitor cells has been recently demonstrated [38]. To determine whether nucleolin-mediated Stat1 nuclear translocation is induced in a stage specific manner during myeolopoiesis, we performed experiments using primary human bone marrow-derived CD34-positive cells. Cells were stimulated with CSF-1 to induce differentiation to the monocyte/macrophage specific unilineage [41]. Differentiation was monitored by expression of specific markers in FACS analysis. Though both, nucleolin and Stat1 were constantly expressed over the whole process of CD34^+^ cells differentiation, we observed nucleolin-Stat1 binding and nuclear translocation only at the stage of monocyte-to-macrophage differentiation; nucleolin silencing impaired Stat1 nuclear accumulation (Fig. 8A). To further investigate the stage-dependent specificity of this effect, we triggered PLB-985 cells to differentiate to neutrophils and analyzed the nucleolin-Stat1 binding in pull-down assays. A high level of Stat1 expression was detected in these cells, however, no binding of Stat1 with nucleolin was observed (Fig. 8B).

To investigate functional consequences of this association for the monocyte-to-macrophage differentiation process, we analyzed the expression of genuine macrophage specific markers after nucleolin silencing. As shown in Fig. 8C, in THP-1 cells, the expression of CD11b and CD36 was significantly decreased by about 50% in Nclsi- compared with control cells. The revealed dependency of the scavenger receptor CD36 on nucleolin is of special interest, because it has been reported recently, that CD36 serves as a specific Stat1 target gene regulating CD36-directed foam cell formation in macrophages [42].

Collectively, these data indicate that nucleolin-Stat1 interaction is specific to the monocyte-to-macrophage step and that intact nucleolin is required to promote the monocytic scavenger receptor CD36.

### Stat1 Nuclear Import Requires the Nuclear Localization Signal of Nucleolin

**Figure 9:**
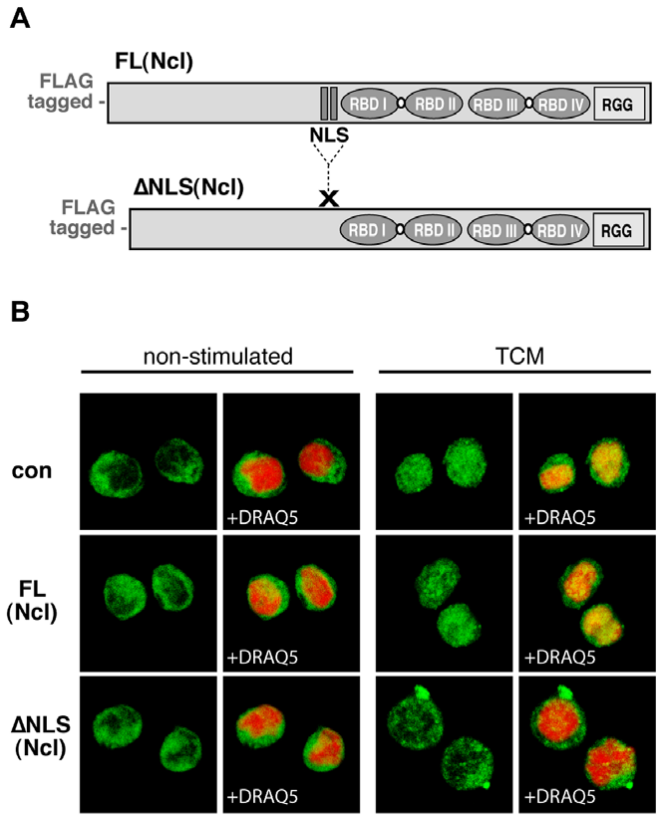
NLS sequence of nucleolin is necessary for the nuclear translocation of Stat1. (A) For infection of monocytic murine M1 cells, a full-length (FL) and a NLS sequence deleted (ΔNLS) nucleolin cDNA from murine origin were used to generate lentiviral particles. (B) Three days post infection with FL(Ncl), ΔNLS(Ncl), or empty lentiviruses (con), M1 cells were stimulated for differentiation with TCM or left non-stimulated. Cells were fixed and permeabilized, and stained for Stat1 (monoclonal anti-Stat1 antibody and corresponding Alexa488-coupled secondary antibody, green colour). Simultaneous DNA labelling with DRAQ5 (red colour) was performed to visualize the nuclear compartment. The translocation of Stat1 into the nucleus was analyzed with a Leica TCS-SP2 AOBS confocal microscope.

Nucleolin shuttles between the cytoplasm and the nucleus, and its NPC-related carrier function for karyophilic proteins depends on the intact NLS [18]–[20]. Our next question was to investigate a role of the nucleolin NLS for the nucleolin-transmitted Stat1 nuclear transport in hematopoietic cells. We used lentiviral gene transfer to overexpress in a dominant-negative fashion a NLS deletion mutant form of nucleolin (Fig. 9A). The subcellular localization of Stat1 was monitored by confocal microscopy; cell nuclei were defined using specific nuclear markers. As shown in Fig. 9B, non-differentiating cells showed bright cytoplasmic staining for Stat1, in contrast to differentiating cells infected with control virus or expressing full-length nucleolin. In these cells, Stat1 accumulated in the nucleus. Stat1 remained almost exclusively cytoplasmic only in the NLS-mutant expressing cells stimulated for differentiation. Just a faint nuclear Stat1 staining was occasionally visible, that reflected most likely a low level of non-competed, wild type nucleolin transmitted transport. These data indicate a requirement of the NLS sequence for the nucleolin-directed Stat1 nuclear transport in hematopoietic cells.

## Discussion

In this study, we identified a novel molecular mechanism for Stat1 nuclear transport. This finding represents an alternative pathway to the already reported Stat1 nuclear translocation mechanisms, which is mediated by the shuttle protein nucleolin. The NLS sequence of nucleolin is necessary for the correct import into the nuclear compartment. The important result of our experiments is that this mechanism is induced in a stage-specific manner along the monocyte/macrophage pathway. Nucleolin deficiency is associated with impaired expression of monocyte differentiation markers, one of which, the scavenger receptor CD36, is a Stat1 target gene and plays an important role in pathophysiological events related to lipid uptake and inflammation.

Many different mechanisms orchestrate the myeloid developmental program, including cooperative gene regulation, protein-protein interactions, and induction of cell cycle arrest. We addressed a role of nucleolin in cell differentiation and identified the transcription factor Stat1 as a novel and specific binding partner of nucleolin in hematopoietic cells. Increasing evidence point to multiple functions of Stat1 in myeloid cells mediating inflammatory, proapoptotic and antiproliferative events [2], [43], [44]. These studies suggest that Stat1 may be an early transcription factor activated during the monocyte maturation process upon migration into extravascular tissues. They further indicate that Stat1 does not trigger *per se* the differentiation process but rather is a part of a developmental program leading to the regulation the transcription of genes specific of mature macrophages. Our findings are along these lines of evidence. We observed the formation of the Stat1-nucleolin complex and its nuclear translocation in the later phase of the monocyte-to-macrophage differentiation. Our results support the studies of others reporting Stat1 constitutive activation at day five of monocyte cultivation [2], as well as changes in activation and DNA binding capacity of LIL-Stat during monocyte maturation [45]. These data suggest that the effects of Stats are tightly controlled according to the status of cell differentiation. The underlying molecular events are, however, sparsely explored.

Specificity of Stat-mediated cellular reactions is a complex multistep process that explains the variety of Stat-mediated cellular functions. This molecular machinery includes the primary stimuli, activation of specific receptors and transactivation of co-receptors, a cross-talk of intracellular signaling pathways and finally, Stat nuclear translocation and DNA binding. Our study provides evidence that Stat1 nuclear transport and DNA binding in a stage-specific manner along the monocyte/macrophage pathway is mediated by the shuttle protein nucleolin. Our results also suggest that nucleo-cytoplasmic shuttle may play a significant role in the modulation of gene expression that occur during monocyte differentiation. The revealed requirement of nucleolin for Stat1 nuclear translocation was surprising, since the molecular mechanisms of Stat1 nuclear transport have been extensively studied before. Thus, Stat1 can associate with specific transport factors of the importin family [46]-[48] or directly interact with the NPC [36]. Also an unusual nuclear import signal that specifically regulates the nuclear entry of tyrosine-phosphorylated dimeric Stat1, termed dimer-specific NLS (dsNLS), was identified [49]. Our findings add to these schemes a novel mechanism for Stat1 nuclear transport, which involves NLS sequence of nucleolin. NLS containing proteins are transported into the nucleus by importins. The classic mono- or bipartite NLS typically contains a cluster of basic residues that is recognized and bound by the NLS receptor importin-α, which is further associated through a separate domain with importin-β [50], [51]. Although it cannot be excluded that the nucleolin-Stat1 complex interacts with importins, our cross-linking experiments do not support this implication. Nucleolin, especially the aminoterminal third, is highly phosphorylated [52], [53], and the efficiency of nucleolin shuttling depends on its phosphorylation state [54]. There is a direct link between the grade of nucleolin phosphorylation and the function of NLS for the nuclear membrane passage through the NPC [18], [19]. Therefore, phosphorylation/dephosphorylation reactions might be a regulatory element of nucleolin localization. Stat1 phosphorylation as a result of its activation upon monocyte differentiation has been demonstrated [27], [30], [55]. Though we confirmed these observations of others, no specific requirement for phosphorylated Stat1 for nucleolin binding was determined (data not shown). Our experiments with INFγ suggest that Stat1 phosphorylation of itself is not sufficient for Stat1 nuclear transport via nucleolin. In agreement with this observation, nuclear translocation of other Stats independently of tyrosine phosphorylation was recently documented [56]. These intriguing issues need further studies.

Nuclear transport mechanisms play a fundamental role in regulating the activity of transcription factors [57]. We provide evidence that nucleolin, beyond mediating Stat1 import into the nucleus, is a part of the Stat1-DNA binding complex. Transcription factors can act as multiprotein complexes, whose components may be involved in different aspects of transcriptional regulation. Adaptor proteins and co-activators may serve for bridging the specific transcription factor to the basic transcription machinery or to facilitate the contact with histone acetylases and deacetylases, which are required for the chromatin remodeling [22], [58]. Interestingly, the N-terminal portion of nucleolin can bind to DNA and histone H1 [59] and the C-terminus shows a helicase activity [60]. Both could be responsible for the capacity of nucleolin to remodel the chromatin structure, an important process for the activation or repression of gene expression [61].

Our results show that association and nuclear translocation of Stat1 and nucleolin is specific for the stage of monocyte/macrophage differentiation. This association was found in different human and murine cell lines and primary cells of monocytic origin including hematopoietic progenitor cells stimulated for monocyte-to-macrophage differentiation. Though being expressed, Stat1 and nucleolin did not associate when cells were stimulated to differentiate to neutrophils and in cells of non-myeloid lineages. Furthermore, in our experiments using nucleolin transgenes we show that IFNγ-induced Stat1 nuclear translocation does not utilize nucleolin and in particular its NLS sequence. Our data support the possibility that the nucleolin-mediated Stat1 nuclear transport might represent a specialized pathway for the time-coordinated control of Stat1-regulated gene expression in myelopoiesis. From a clinical perspective, it would be interesting to identify factors, which specifically induce this pathway *in vivo*. Our results indicate that this pathway is most likely regulated via the CSF-1/M-CSFR system. Conversely, we need to know whether a paracrine or an autocrine mechanism is involved.

With the exception for some IFN-stimulated genes, the cellular genes dependent on cytokine-activated Stat proteins are poorly defined. Recent studies point to a novel function of Stat1 in the pathogenesis of numerous diseases beyond tumorigenesis and host defenses, such as atherosclerosis and other cardiovascular disorders [62], [63]. It has been shown that myocardial ischemia and reperfusion induced a rapid activation of Stat1 [63], [64]. This functional outcome from Stat1 activation was related to promoting apoptotic cell death upon ischemia/reperfusion injury. A critical role of Stat1 as a sensor responding to cellular stress was further demonstrated *in vitro* and *in vivo* in endoplasmic reticulum stress-induced macrophage apoptosis and atherosclerotic plaque progression [65]. Stat1 may have an additional role in the early lesions that is independent of macrophage cell death but rather related to regulation of CD36 scavenger receptor expression and foam cell formation [42]. These observations may explain, at least in part, why Stat1 deficiency in apolipoprotein E-/- mice blocks foam cell formation and early lesion development. Our results suggest that nucleolin-directed Stat1 nuclear transport during the course of macrophage maturation might represent a specific pathway to regulate CD36 expression. Thus, we observed that nucleolin silencing resulted in abrogation of CD36 expression. The regulation of CD36 by Stat1 may be important in other pathophysiological events involving CD36-dependent lipid uptake and inflammation, such as diabetes mellitus and the metabolic syndrome. Therefore, Stat1 inhibition could represent a target to reduce inflammation and to prevent progression of these diseases. Elucidation of the underlying molecular mechanisms of Stat1 regulation could lead to enhanced understanding of these physiological and pathophysiological processes. Our study indicates Stat1-nucleolin interference as one of these mechanisms.

## Materials and Methods

### Materials

High-quality commercial grade chemicals were purchased from Sigma (St. Louis, MO), Merck (Darmstadt, Germany), and Roth (Karlsruhe, Germany). Chemiluminescent signal enhancer was obtained from NEN™ Life Science Products, Inc. (Boston, MA). The far-red fluorescent DNA dye DRAQ5 was from Biostatus Limited Ltd. (Shepshed, UK). Aqua-Poly/Mount mounting media was purchased from Polysciences, Inc. (Warington, PA). Oligonucleotides were from Santa Cruz Biotechnology, Inc. (Santa Cruz, CA).

### Antibodies

Monoclonal antibodies for Stat1 protein were from BD Transduction Laboratories (Lexington, KY). Monoclonal anti-nucleolin antibodies were purchased from Medical and Biological Laboratories, Co, Ltd. (Nagoya, Japan) and from Santa Cruz Biotechnology (sc-8031). Polyclonal antibodies for Stat1 (sc-345), M-CSFR, histone H1 and β-tubulin were from Santa Cruz Biotechnology. Polyclonal anti-phospho-M-CSFR (Tyr723) antibody was obtained from Cell Signaling Technology, Inc. (Danvers, MA). FITC- or PE-conjugated specific antibodies or isotype-matched controls for FACS analysis were obtained from Immunotech (Marseilles, France), BD Pharmingen (San Diego, CA), and Serotec (Oxford, UK). Fluorescent Alexa 488- and Alexa 594-conjugated secondary antibodies were from Molecular Probes, Inc. (Eugene, OR). Horseradish peroxidase-conjugated secondary antibodies were purchased from Jackson ImmunoResearch Laboratories (West Grove, PA) and Santa Cruz, Inc.

### Cell Culture

Peripheral blood mononuclear cells (PBMC) were isolated from healthy volunteers using Biocoll Separation Solution (Biochrom KG Seromed, Berlin, Germany) according to the standard protocol. After density gradient centrifugation, pure monocyte fraction (purity approx. 92%) was obtained by using an indirect magnetic labeling system (Monocyte Isolation Kit II, Miltenyi Biotec Inc., Auburn, CA), as advised by the manufacturers. Monocytes were cultured in RPMI 1640 medium (Biochrom) supplemented with 10% fetal bovine serum, 2 mM L-glutamine, 100 U/ml penicillin and 100 µg/ml streptomycin. For stimulation, 50 ng/ml CSF-1 (M-CSF) (R&D Systems, Minneapolis, MN) was used for indicated time points.

The human U937 promyelocytic cell line (American Type Culture Collection (ATTC), Manassas, VA) and the human myeloid leukaemia cell line THP-1 (German Resource Centre for Biological Material (DSMZ), Braunschweig, Germany) were cultured in supplemented RPMI 1640 medium. Induction of monocyte-to-macrophage differentiation was performed by cell stimulation with 2–20 nM PMA (Sigma) for different time points.

M1 mouse myeloid leukemia cells (DSMZ) were cultured in Dulbecco's modified Eagle's medium (DMEM) (Biochrom) supplemented with 10% fetal bovine serum, 2 mM L-glutamine, 100 U/ml penicillin and 100 µg/ml streptomycin. Monocyte-macrophage differentiation was induced by cell stimulation with conditioned media from THP-1 cells (TCM) as described [66].

The human myeloid leukemia cell line PLB-985 (DSMZ) was cultured in supplemented RPMI 1640 medium. Induction of neutrophil differentiation was performed by cell stimulation with 1.25% DMSO for 5 days.

Source of peripheral blood CD34+ cells were leukapheresis products from healthy donors after stem cell mobilization with recombinant granulocyte-colony stimulating factor (G-CSF). For isolation of CD34+ cells, the CliniMACS system (Miltenyi Biotec, Bergisch Gladbach, Germany) was used. The purity of the isolated cells for CD34+ was >99%. The majority of the CD34+ cell population (∼80%) was double positive for the common leukocyte antigen CD45 and the stem cell marker CD133. Cells were cultivated in StemSpanRSFEM (Serum-free medium for expansion and culture of hematopoietic cells) from StemCell Technologies Inc, supplemented with 100 ng/ml Stem Cell Factor (Cell Systems), 50 ng/ml Flt3 ligand (Cell Systems), 20 ng/ml TPO (thrombopoietin, Peprotech EC Ltd). Differentiation to the monocytic lineage was initiated by 10 ng/ml human CSF-1 (M-CSF) (R&D Systems).

### Plasmid Construction

The nucleolin constructs (ΔC(Ncl) aa284-aa707, and C(Ncl) aa538-aa707) were generated from a human nucleolin cDNA clone [53] and subcloned into the pSG5 vector (Stratagene, La Jolla, CA). To generate the GST fusion proteins, the corresponding constructs were cloned into the bacterial expression vector pGEX-2T (Amersham Pharmacia Biotec Inc.) and expressed after Isopropyl-1-thio-b-D-galactopyranoside (IPTG) induction in DH5α E.coli strain.

Mouse nucleolin cDNA was obtained from the American Type Culture Collection (ATCC; Manassas, VA). The sequence encoding full-length mouse nucleolin was subcloned into pMT/BiP/V5-His expression vector (Invitrogen, Carlsbad, CA). The coding sequence for a FLAG epitope was introduced at the N-terminus of nucleolin (pMT/BiP-N-FLAG-m-ncl) using QuickChange® site-directed mutagenesis kit (Stratagen, La Jolla, CA). pMT/BiP-N-FLAG-m-ncl plasmid served as a template for further cloning procedures. Constructs encoding N-terminus FLAG-tagged wild-type mouse nucleolin and a mutant, in which the NLS (encoded by nt 841 to 897) was deleted (ΔNLS) were constructed and cloned in pcDNA3.1(+) vector (Invitrogen) using standard PCR-mediated cloning procedures. For overexpression of mouse nucleolin, lentivirus transfer vector pWPTS-adapter was generated from the pWPTS-GFP vector by BamHI and SalI cloning duplex. Full length mouse nucleolin cDNA and NLS nucleolin mutant were transferred from pcDNA3.1(+) into pWPTS-adapter in SalI and SpeI sites. Final construct were designated as pWPTS-FL(Ncl) and pWPTS-ΔNLS(Ncl) accordingly.

For nucleolin silencing, the target sites in human nucleolin mRNA (Acc. #NM005381) for RNAi were determined using the siRNA Selection Server (http://jura.wi.mit.edu/bioc/siRNAext/home.php)41 and designed as oligonucleotides encoding short hairpin RNAs (shRNAs). The following complimentary sequences were selected:

Nuc 64

G AGG TAG AAG AAG ATA GTT

Nuc 719

A CGC TAA AGA AGC TTT AAT

For cloning in pLVTHM vector, the following duplexes were used:

Nuc 64

CGCGTCCCCGAGGTAGAAGAAGATAGTTTTCAAGAGAAACTATCTTCTTCTACCTCTTTTTGGAAAT

CGATTTCCAAAAAGAGGTAGAAGAAGATAGTTTCTCTTGAAAACTATCTTCTTCTACCTCGGGGA

Nuc 719

CGCGTCCCCACGCTAAAGAAGCTTTAATTTCAAGAGAATTAAAGCTTCTTTAGCGTTTTTTGGAAAT

CGATTTCCAAAAAACGCTAAAGAAGCTTTAATTCTCTTGAAATTAAAGCTTCTTTAGCGTGGGGA

For downregulation of nucleolin expression by a lentivirus containing nucleolin-specific siRNA, pLVTH-Nclsi plasmid was generated by ligation of the oligonucleotide duplex Nclsi in MluI and ClaI sites of pLVTHM (Tronolab, Switzerland). Nuc 64 showed more extended silencing property and was therefore selected for the next experiments.

For M-CSFR overexpression, the lentivirus pDEST-lenti transfer vector was generated by blant ligation Gateway Cassete rfa-verB (Invitrogen) in PmeI and SmaI sates of the pLV-tRKRAB-Red vector (Tronolab). Entry clones for the transfer of the M-CSFR were produced by cloning the PCR products in the pENTR/D TOPO plasmid (Invitrogen). For M-CSFR overexpression, pEXPR clones were generated by site-specific recombination between pDEST-lenti and pENTR/D TOPO-M-CSFR by Gateway LR Clonase Enzyme mix (Invitrogen).

### Lentiviral Vector Production and Cell Infection

For experiments with M-CSFR constructs, pCMV-dR8.74, pMD.2G (Tronolab) and pEXPR plasmids were co-transfected (using ratio pLVTHM∶pCMV-dR8.74∶pMD2G = 3∶2∶1) into 293T cells by PerFectin transfection reagent (Genlantis, San Diego, CA) as recommended by manufacturer. After 48 h post transfection, the viral particles containing cell supernatants were harvested, filtered, concentrated, and stored at −70°C for future use. pLVTH-Nclsi, pWPTS-FL(Ncl), pWPTS-ΔNLS(Ncl) vectors were used instead pEXPR for nucleolin silencing or overexpression as stated above.

For infection, 0.5–0.7×10^6^ cells/0.6 ml in the presence of 16 µg polybrene and 0.4 ml virus stock were centrifuged (1,000×g, 30 min, RT) and incubated at 37°C with 5% CO_2_ for 4 to 5 hrs. Cells were transferred into 2 ml for overnight and then into 6 ml fresh RPMI medium for further cultivation.

### Quantitative RT-PCR Analysis of Nucleolin

The total RNA samples was isolated from THP-1 cells using QIAGEN QiaSpin miniprep kit and DNAse kit (QIAGEN, Hilden, Germany), and real-time quantitative RT-PCR for nucleolin mRNA was performed on a TaqMan ABI 7700 Sequence Detection System (Applied Biosystems, Foster City, CA, USA). GAPDH was used as a reference gene. The following oligonucleotide primers and probes were used: GAPDH, 5′-GAAGGTGAAGGTCGGAGTC-3′ (sense), 5′-GAAGATGGTGATGGGATTC-3′ (antisense), 6-FAM-CAAGCTTCCCGTTCTCAGCC-TAMRA (probe); Nucleolin, 5′- TCGCGAAGGCAGGTAAAAA-3′ (sense), 5′- CGACCTCTTCTCCACTGCTATCA-3′ (antisense), 5′- 6-FAM-AAGGTGACCCCAAGAAAATGGCTCCTC-TAMRA (probe).

### Cell Lysis and Immunoblotting

Non-stimulated cells were washed twice with PBS [phosphate buffered saline], stimulated, adherent cells were detached by adding 5 mM EDTA [ethylene diamine tetramine acetate] in PBS. Cells were washed twice with ice cold PBS, resuspended in lysis buffer (20 mM Tris-HCl, pH 8.0, 138 mM NaCl, 10% glycerol, 2 mM EDTA, 1% Triton X-100, and protease inhibitors 1 mM PMSF [phenylmethylsulfonyl fluoride], 10 µg/ml aprotinin, 10 µg/ml leupetin, 0.3 mM sodium orthovanadate), and put on ice for 10 min. Lysates were clarified by centrifugation at 13,000 rpm for 10 min. Western blotting was performed as described elsewhere [67].

### Fusion Protein Precipitation Assay (Pull Down Assay)

Nucleolin and/or Stat1 GST fusion proteins immobilized on glutathione- agarose beads (Sigma) were used for affinity precipitation. Cell lysates containing 800 to 1,500 µg protein were incubated for 1 hr at RT or overnight at 4°C with immobilized GST fusion protein. GST- matrix was used as control affinity matrix. Precipitates were washed three times with Tris-buffed saline containing 0.1% Tween20 (TBS-T). Precipitated proteins were eluted with Laemmli sample buffer containing 20 mM dithiotreitol (DTT), and were used for SDS-PAGE and Immunoblotting with corresponding antibodies.

### Immunoprecipitation and Cross-Linking

Immunoprecipitation studies were performed as described [67], [68]. For cross-linking, 2 mM of the bifunctional chemical cross-linker bis(sulfosuccinimidyl) suberate (BS^3^, Pierce, Rockford, IL) was added to cytosolic fractions for 30 min at RT. The reaction was stopped by adding Tris to a final concentration of 50 mM and incubated for further 15 min. Cross-linked samples were used for immunprecipitation and Western blotting.

### Nuclear Extract Preparation

The cell pellet of approx. 3.5×10^7^ cells was washed, resuspended in 1 ml hypotonic buffer (10 mM HEPES pH 7.9, 1.5 mM MgCl_2_, 10 mM KCl, 0.5 mM DTT) and incubated on ice for 30 min. After sonication and centrifugation (600×g, 5 min, 4°C), the pellet was resuspended in 600 µl 0.25 M sucrose in buffer A (50 mM Tris-HCl, pH 7.4, 5 mM MgSO_4_, 2 mM DTT), laid over 400 µl 0.25 M sucrose in buffer A and centrifuged (700×g, 7 min, 4°C). The pellet was resuspended in 6 ml 1.4 M sucrose in buffer A and laid between 4 ml 2.2 M sucrose and 2 ml 0.25 M sucrose in buffer A. After ultracentrifugation (100,000×g for 45 min at 4°C, Beckmann Sw40Ti), the pure nuclei were resuspended in 40 µl of a low salt buffer (20 mM HEPES, pH 7.9, 25% glycerol, 20 mM KCl, 0.2 mM EDTA, 0.5 mM DTT). 30 µl high salt buffer (20 mM HEPES, pH 7.9, 25% glycerol, 1.5 mM MgCl_2_, 800 mM KCl, 0.2 mM EDTA, 0.5 mM DTT), were added and incubated on ice for 20 min, followed by centrifugation at 13,000×g (5 min, 4°C). The supernatant was dialyzed against 20 mM HEPES, pH 7.9, 20% glycerol, 100 mM KCl, 0.2 mM EDTA, 0.5 mM DTT).

For crude nuclear extracts, the cell pellet of 1.5×10^7^ monocytes was resuspended in 400 µl ice cold extract buffer (20 mM HEPES, pH 7.9, 0.1 mM EDTA, 0.1 mM EGTA, 1 mM DTT) containing 10 mM KCl, placed on ice for 20 min and lysed in 0.5% Nonidet P-40. After centrifugation, the pellet was resuspended in 50 µl ice cold extract buffer containing 20% glycerol and 400 mM NaCl and incubated at 4°C for 30 min. After centrifugation, crude nuclear extracts were microdialyzed against PBS, used immediately or stored at −80°C.

### Electric Mobility Shift Assay (EMSA)

Electrophoretic mobility shift assays were performed as described previously [69]. Binding reaction was performed for 30 min at 25°C with 2–4 µg of whole cell extract. For antibody-supershift and competition analysis protein extracts were pre-incubated with 3 µg of Stat1 polyclonal or nucleolin monoclonal antibodies for 30 min on ice. For oligonucleotide competition 5- fold excess of unlabeled oligonucleotides were added to the reaction. The following double-stranded oligonucleotides were used:

GAS sense, 5′-CATGTTATGCATATTCCTGTAAGTG-3′

GAS anti-sense, 5′-CATGCACTTACAGGAATATGCATAA-3′

H2K sense 5′-GATCCAGGGCTGGGGATTCCCCATCTCCACAGG-3′

H2K anti-sense 5′-GTCCCGACCCCTAAGGGGTAGAGGTGTCCCTAG-3′

### Immunofluorescence Confocal Microscopy

Suspension cells, primary human blood monocytes or monocytic cells (THP-1, U937, M1, CD34+), were pelleted on microscope slides by using a cytospinner (Hettich, Tuttlingen Germany, 200×g, 7 min). Stimulated, adherent cells were seeded and cultured on glass coverslips. Cells were fixed, stained and mounted as described by us previously [67]. Fc receptors were blocked with Fc receptor blocking reagents (Miltenyi Biotech or BD Biosciences). DNA staining was performed with far-red fluorophore DRAQ5 (Biostatus Limited Ltd., 1∶100, 15 min at RT) or with DAPI [4′,6-Diamidin-2′-phenylindoldihydrochlorid] (Invitrogen, 300 nM in PBS, 5 min at RT).

### FACS Analysis

Phenotypic expression of CD11b, CD36, and CD206 was quantified by FACS (FACscan; Becton Dickinson, Heidelberg, Germany). Briefly, adherent cells were detached with 5 mM EDTA in PBS. Cells were collected by centrifugation, incubated with Fc receptor blocking reagents and stained with the corresponding FITC- or PE-conjugated specific antibody or the isotype matched control. For quantitative analysis of nucleolin, cells were fixed with 2% paraformadehyde in PBS for 15 min at RT and permeabilized with an ice-cold mixture of methanol and acetone (1∶1) at −20°C for 20 min. After incubation with monoclonal anti- nucleolin IgG for 30 min at RT, the Alexa488-conjugated secondary antibody was added for further 15 min at RT. Measurement of mean fluorescence intensity (MFI) and analysis of data were done using Cell Quest Software. 10,000 events per sample were collected.

## References

[1] Friedman AD (2002). Transcriptional regulation of granulocyte and monocyte development.. Oncogene.

[2] Coccia EM, Del Russo N, Stellacci E, Testa U, Marziali G (1999). STAT1 activation during monocyte to macrophage maturation: role of adhesion molecules.. Int Immunol.

[3] Ginisty H, Sicard H, Roger B, Bouvet P (1999). Structure and functions of nucleolin.. Journal Of Cell Science.

[4] Srivastava M, Pollard HB (1999). Molecular dissection of nucleolin's role in growth and cell proliferation: new insights.. Faseb J.

[5] Tuteja R, Tuteja N (1998). Nucleolin: a multifunctional major nucleolar phosphoprotein.. Crit Rev Biochem Mol Biol.

[6] Caizergues-Ferrer M, Mariottini P, Curie C, Lapeyre B, Gas N (1989). Nucleolin from Xenopus laevis: cDNA cloning and expression during development.. Genes Dev.

[7] Gillet G, Michel D, Crisanti P, Guerin M, Herault Y (1993). Serum factors and v-src control two complementary mitogenic pathways in quail neuroretinal cells in culture.. Oncogene.

[8] Bose S, Basu M, Banerjee AK (2004). Role of nucleolin in human parainfluenza virus type 3 infection of human lung epithelial cells.. J Virol.

[9] de Verdugo UR, Selinka HC, Huber M, Kramer B, Kellermann J (1995). Characterization of a 100-kilodalton binding protein for the six serotypes of coxsackie B viruses.. J Virol.

[10] Saxena A, Rorie CJ, Dimitrova D, Daniely Y, Borowiec JA (2006). Nucleolin inhibits Hdm2 by multiple pathways leading to p53 stabilization.. *Oncogene*.

[11] Mi Y, Thomas SD, Xu X, Casson LK, Miller DM (2003). Apoptosis in leukemia cells is accompanied by alterations in the levels and localization of nucleolin.. J Biol Chem.

[12] Hirano K, Miki Y, Hirai Y, Sato R, Itoh T (2005). A multifunctional shuttling protein nucleolin is a macrophage receptor for apoptotic cells.. J Biol Chem.

[13] Pasternack MS, Bleier KJ, McInerney TN (1991). Granzyme A binding to target cell proteins. Granzyme A binds to and cleaves nucleolin in vitro.. J Biol Chem.

[14] Borer RA, Lehner CF, Eppenberger HM, Nigg EA (1989). Major nucleolar proteins shuttle between nucleus and cytoplasm.. Cell.

[15] Hovanessian AG (2006). Midkine, a cytokine that inhibits HIV infection by binding to the cell surface expressed nucleolin.. Cell Res.

[16] Dumler I, Stepanova V, Jerke U, Mayboroda OA, Vogel F (1999). Urokinase-induced mitogenesis is mediated by casein kinase 2 and nucleolin.. Curr Biol.

[17] Semenkovich CF, Ostlund RE,, Olson MO, Yang JW (1990). A protein partially expressed on the surface of HepG2 cells that binds lipoproteins specifically is nucleolin.. Biochemistry.

[18] Schmidt-Zachmann MS, Nigg EA (1993). Protein localization to the nucleolus: a search for targeting domains in nucleolin.. J Cell Sci.

[19] Creancier L, Prats H, Zanibellato C, Amalric F, Bugler B (1993). Determination of the functional domains involved in nucleolar targeting of nucleolin.. Mol Biol Cell.

[20] Bouvet P, Diaz JJ, Kindbeiter K, Madjar JJ, Amalric F (1998). Nucleolin interacts with several ribosomal proteins through its RGG domain.. J Biol Chem.

[21] Yang TH, Tsai WH, Lee YM, Lei HY, Lai (1994). Purification and characterization of nucleolin and its identification as a transcription repressor.. Mol Cell Biol.

[22] Ying GG, Proost P, van Damme J, Bruschi M, Introna M (2000). Nucleolin, a novel partner for the Myb transcription factor family that regulates their activity.. J Biol Chem.

[23] Hanakahi LA, Dempsey LA, Li MJ, Maizels N (1997). Nucleolin is one component of the B cell-specific transcription factor and switch region binding protein, LR1.. Proc Natl Acad Sci U S A.

[24] Mui AL, Wakao H, Kinoshita T, Kitamura T, Miyajima A (1996). Suppression of interleukin-3-induced gene expression by a C-terminal truncated Stat5: role of Stat5 in proliferation.. Embo J.

[25] Meinke A, Barahmand-Pour F, Wohrl S, Stoiber D, Decker T (1996). Activation of different Stat5 isoforms contributes to cell-type-restricted signaling in response to interferons.. Mol Cell Biol.

[26] Nakajima K, Yamanaka Y, Nakae K, Kojima H, Ichiba (1996). A central role for Stat3 in IL-6-induced regulation of growth and differentiation in M1 leukemia cells.. Embo J.

[27] Dimberg A, Nilsson K, Oberg F (2000). Phosphorylation-deficient Stat1 inhibits retinoic acid-induced differentiation and cell cycle arrest in U-937 monoblasts.. Blood.

[28] Nagamura-Inoue T, Tamura T, Ozato K (2001). Transcription factors that regulate growth and differentiation of myeloid cells.. Int Rev Immunol.

[29] Barahmand-Pour F, Meinke A, Kieslinger M, Eilers A, Decker T (1996). A role for STAT family transcription factors in myeloid differentiation.. Curr Top Microbiol Immunol.

[30] Eilers A, Decker T (1995). Activity of Stat family transcription factors is developmentally controlled in cells of the macrophage lineage.. Immunobiology.

[31] Levy DE, Darnell JE, (2002). Stats: transcriptional control and biological impact.. Nat Rev Mol Cell Biol.

[32] Tran EJ, Wente SR (2006). Dynamic nuclear pore complexes: life on the edge.. Cell.

[33] Zhong H, Takeda A, Nazari R, Shio H, Blobel G (2005). Carrier-independent nuclear import of the transcription factor PU.1 via RanGTP-stimulated binding to Nup153.. J Biol Chem.

[34] Xu L, Alarcon C, Col S, Massague J (2003). Distinct domain utilization by Smad3 and Smad4 for nucleoporin interaction and nuclear import.. J Biol Chem.

[35] Meyer T, Vinkemeier U (2004). Nucleocytoplasmic shuttling of STAT transcription factors.. Eur J Biochem.

[36] Marg A, Shan Y, Meyer T, Meissner T, Brandenburg M (2004). Nucleocytoplasmic shuttling by nucleoporins Nup153 and Nup214 and CRM1-dependent nuclear export control the subcellular distribution of latent Stat1.. J Cell Biol.

[37] Tu X, Baffa R, Luke S, Prisco M, Baserga R (2003). Intracellular redistribution of nuclear and nucleolar proteins during differentiation of 32D murine hemopoietic cells.. Exp Cell Res.

[38] Grinstein E, Du Y, Santourlidis S, Christ J, Uhrberg M (2007). Nucleolin regulates gene expression in CD34-positive hematopoietic cells.. J Biol Chem.

[39] Reich NC, Liu L (2006). Tracking STAT nuclear traffic.. Nat Rev Immunol.

[40] Martinet W, Schrijvers DM, Kockx MM (2003). Nucleofection as an efficient nonviral transfection method for human monocytic cells.. Biotechnol Lett.

[41] Rosa A, Ballarino M, Sorrentino A, Sthandler O, De Angelis FG (2007). The interplay betwen the master transcription factor PU.1 and miR-424 regulates human monocyte/macrophage differentiation.. PNAS.

[42] Agrawal S, Febbraio M, Podrez E, Cathcart MK, Stark GR (2007). Signal transducer and activator of transcription 1 is required for optimal foam cell formation and atherosclerotic lesion development.. Circulation.

[43] Kalliolias GD, Ivashkiv LB (2008). IL-27 activates human monocytes via STAT1 and suppresses IL-10 production but the inflammatory functions of IL-27 are abrogated by TLRs and p38.. J Immunol.

[44] Galdiero M, Vitiello M, D'Isanto M, Raieta K, Galdiero E (2006). STAT1 and STAT3 phosphorylation by porins are independen of JAKs but are dependent on MAPK pathway and plays a role in U937 cells production of interleukin-6.. Cytokine.

[45] Tuyt LM, Bregman K, Lummen C, Dokter WH, Vellenga E (1998). Differential binding activity of the transcription factor LIL-STAT in immature and differentiated normal and leukemic myeloid cells.. Blood.

[46] Fagerlund R, Melen K, Kinnunen L, Julkunen I (2002). Arginine/lysine-rich nuclear localization signals mediate interactions between dimeric STATs and importin alpha 5.. J Biol Chem.

[47] McBride KM, Banninger G, McDonald C, Reich NC (2002). Regulated nuclear import of the STAT1 transcription factor by direct binding of importin-alpha.. Embo J.

[48] Sekimoto T, Imamoto N, Nakajima K, Hirano T, Yoneda Y (1997). Extracellular signal-dependent nuclear import of Stat1 is mediated by nuclear pore-targeting complex formation with NPI-1, but not Rch1.. Embo J.

[49] Meyer T, Begitt A, Lodige I, van Rossum M, Vinkemeier U (2002). Constitutive and IFN-gamma-induced nuclear import of STAT1 proceed through independent pathways.. Embo J.

[50] Jans DA, Xiao CY, Lam MH (2000). Nuclear targeting signal recognition: a key control point in nuclear transport?. Bioessays.

[51] Weis K (2003). Regulating access to the genome: nucleocytoplasmic transport throughout the cell cycle.. Cell.

[52] Jordan G (1987). At the heart of the nucleolus.. Nature.

[53] Srivastava M, Fleming PJ, Pollard HB, Burns AL (1989). Cloning and sequencing of the human nucleolin cDNA.. FEBS Lett.

[54] Schwab MS, Dreyer C (1997). Protein phosphorylation sites regulate the function of the bipartite NLS of nucleolin.. Eur J Cell Biol.

[55] Dimberg A, Karehed K, Nilsson K, Oberg F (2006). Inhibition of monocytic differentiation by phosphorylation-deficient Stat1 is associated with impaired expression of Stat2, ICSBP/IRF8 and C/EBPepsilon.. Scand J Immunol.

[56] Liu L, McBride KM, Reich NC (2005). Stat3 nuclear import is independent of tyrosine phosphorylation and mediated by importin-alpha3. Proc Natl Acad Sci.. USA.

[57] Xu L, Massague J (2004). Nucleocytoplasmic shuttling of signal transducers.. Nat Rev Mol Cell Biol.

[58] Chatterjee-Kishore M, van den Akker F, Stark GR (2000). Association of STATs with relatives and friends.. Trends Cell Biol.

[59] Erard MS, Belenguer P, Caizergues-Ferrer M, Pantaloni A, Amalric F (1988). A major nucleolar protein, nucleolin, induces chromatin decondensation by binding to histone H1.. Eur J Biochem.

[60] Tuteja N, Ochem A, Taneja P, Tuteja R, Skopac D (1995). Purification and properties of human DNA helicase VI.. Nucleic Acids Res.

[61] Angelov D, Bondarenko VA, Almagro S, Menoni H, Mongelard F (2006). Nucleolin is a histone chaperone with FACT-like activity and assists remodeling of nucleosomes.. Embo J.

[62] Wincewicz A, Sulkowska M, Rutkowski R, Sulkowski S, Musiatowicz B (2007). STAT1 and STAT3 as intracellular regulators of vascular remodeling.. Eur J Intern Med.

[63] Bolli R, Dawn B, Xuan YT (2003). Role of the JAK-STAT pathway in protection against myocardial ischemia/reperfusion injury.. Trends Cardiovasc Med.

[64] Stephanou A (2004). Role of STAT-1 and STAT-3 in ischemia/reperfusion injury.. J Cell Mol Med.

[65] Lim WS, Timmins JM, Seimon TA, Sadler A, Kolodgie FD (2008). Signal transducer and activator of transcription-1 is critical for apoptosis in macrophages subjected to endoplasmic reticulum stress in vitro and in advanced atherosclerotic lesions in vivo.. Circulation.

[66] Kurata S, Nakajima H (1990). Transcriptional activation of the heme oxygenase gene by TPA in mouse M1 cells during their differentiation to macrophage.. Exp Cell Res.

[67] Dumler I, Kopmann A, Wagner K, Mayboroda OA, Jerke U (1999). Urokinase Induces Activation and Formation of Stat4 and Stat1-Stat2 Complexes in Human Vascular Smooth Muscle Cells.. J Biol Chem.

[68] Kiyan J, Kiyan R, Haller H, Dumler I (2005). Urokinase-induced signaling in human vascular smooth muscle cells is mediated by PDGFR-beta.. Embo J.

[69] Hinz M, Broemer M, Arslan S, Otto A, Mueller E (2007). Signal responsiveness of IkappaB kinases is determined by Cdc37-assisted transient interaction with Hsp90.. J Biol Chem.

